# Investigation and Countermeasures Research of Hospital Information Construction of Tertiary Class-A Public Hospitals in China: Questionnaire Study

**DOI:** 10.2196/41820

**Published:** 2023-01-20

**Authors:** Chang Shu, Yueyue Chen, Huiyuan Yang, Ran Tao, Xiaoping Chen, Jingjing Yu

**Affiliations:** 1 Department of Surgery Tongji Hospital, Tongji Medical College Huazhong University of Science and Technology Wuhan China; 2 Hepatic Surgery Center Tongji Hospital, Tongji Medical College Huazhong University of Science and Technology Wuhan China; 3 Hubei Key Laboratory of Hepato-Pancreatic-Biliary Diseases Tongji Hospital, Tongji Medical College Huazhong University of Science and Technology Wuhan China

**Keywords:** public hospital, hospital information construction, current situation, development, countermeasures

## Abstract

**Background:**

Medical informatization has initially demonstrated its advantages in improving the medical service industry. Over the past decade, the Chinese government have made a lot of effort to complete infrastructural information construction in the medical and health domain, and smart hospitals will be the next priority according to policies released by Chinese government in recent years.

**Objective:**

To provide strategic support for further development of medical information construction in China, this study aimed to investigate the current situation of medical information construction in tertiary class-A public hospitals and analyze the existing problems and countermeasures.

**Methods:**

This study surveyed 23 tertiary class-A public hospitals in China who voluntarily responded to a self-designed questionnaire distributed in April 2020 to investigate the current medical information construction status. Descriptive statistics were used to summarize the current configurations of hospital information department, hospital information systems, hospital internet service and its application, and the satisfaction of hospital information construction. Interviews were also conducted with the respondents in this study for requirement analysis.

**Results:**

The results show that hospital information construction has become one of the priorities of the hospitals’ daily work, and the medical information infrastructural construction and internet service application of the hospitals are good; however, a remarkable gap among the different level of hospitals can be observed. Although most hospitals had built their own IT team to undertake information construction work, the actual utilization rate of big data collected and stored in the hospital information system was not satisfactory.

**Conclusions:**

Support for the construction of information technology in primary care institutions should be increased to balance the level of development of medical informatization in medical institutions at all levels. The training of complex talents with both IT and medical backgrounds should be emphasized, and specialized disease information standards should be developed to lay a solid data foundation for data utilization and improve the utilization of medical big data.

## Introduction

With the accelerating pace of information construction in various countries, human society has gradually entered the information age. The development of information technology has promoted the prosperity of medical informatization [1]. Diversified medical scenarios have been expected to transition from theory to reality to meet the diverse medical needs of the people supported by cutting-edge technologies. Artificial intelligence could provide symptom monitoring, predictive modeling, and decision support through its advanced algorithms and learning capabilities [2,3]. Internet of Things could give rise to many medical apps to achieve remote control and health monitoring, fitness programs, chronic disease, and older adult care [4]. Nature language processing could be a powerful tool to deal with information overload in the health and medical field [5]. Many studies had illustrated the benefit that medical information brings to the patients, doctors, and health policy makers. Northwestern Medicine developed an electronic medical record (EMR)–integrated, patient-reported system to comprehensive assess the in-hospital and out-hospital data of patients with cancer, and the system had yielded perceived usefulness on prognosis management from both patients with cancer and health staffs [6]. Several guidance apps, such as Outpatient Guidance System [7], EasyHos System [8], TR2 system [9], etc, successfully link the data between the EMR console and patients’ mobile phone to optimize the medical examination process and effectively reduce the outpatient burden imposed by wait times.

Over the past decade, the Chinese government have made a lot of effort toward information construction in the medical and health domain. In 2009, the China State Council published *Opinions of the CPC Central Committee and State Council on Deepening the Reform of the Medical and Health System*, which emphasized the importance of information construction in hospital rating systems [10]. In 2016, the *Healthy China 2030 Plan outline* released by the China State Council pointed out that the key direction for information construction should be building a unified authoritative, interconnected population health information platform; promoting the open-sharing, in-depth mining, and wide application of medical and health big data base on the population health information platform; and establishing a cross-department and cross-field health and medical data–sharing mechanism with close cooperation and unified centralization [11]. In 2018, The Hospital Management Research Institute of the National Health Commission issued a new standard to measure the application level of EMR (9 levels ranging from 0 to 8) [12]. According to the survey results in 2019, the average EMR level of tertiary hospitals was over grade 3, which meant that cross-department data exchange was achieved within the hospital [13]. Furthermore, the EMR levels of 34% of tertiary hospitals and 24.3% of secondary hospitals had reached grade 5 or above, meaning that they had at least achieved unified data management and intermediate medical decision support [14]. These results were in line with the Chinese government’s anticipated demand. It can be said that China’s hospitals had achieved the infrastructure of informatization construction in the medical and health domain. In 2020, *Further improve the appointment system and strengthen the construction of smart hospitals* [15] was released by the National Health Commission, which makes a clear priority of smart hospitals that include 3 core aspects: smart medical care, smart service, and smart management. Obviously, medical information construction at this stage is more than transforming paper processes into computer processes. Data inside and outside the hospital should all be collected, analyzed, and utilized. Around the theme of “smart hospitals,” many construction solutions have emerged [16]. EMR quality control and diagnosis-related group or big data diagnosis-intervention packet are addressed by automatic coding technology based on artificial intelligence. Nature language process combined with knowledge graph is still the core technology to build a hospital-specialized disease database, clinical decision support system of a single disease, and scientific research platform. Wi-Fi 6.0 and 5G technology ensure that a hospital’s internal and external network are strict isolated, thereby the security and efficiency of hospital work could be ensured.

Apparently, it is possible to use big data technology to build an intelligent platform for health and medical big data, which could promote the vigorous development of multicenter clinical research and fully stimulate the intrinsic economic value of medical big data. Australia had developed a versatile big data health system for cardiovascular health [17], and South Korea had developed a cancer big data platform for cancer research [18]. However, they both noted that one of their current barrier is data fusion among different institutions with different standards. In China, an intelligence platform for nasopharyngeal carcinoma was built [19], but it only handles the data at 1 hospital. The researchers in this study are staff members of the Chen Xiaoping Academician Workstation of Hepatobiliary and Pancreatic Surgery. The academician workstation is devoted to constructing a big data center for hepatobiliary and pancreatic surgery. Therefore, we conducted this study to investigate the current situation of hospital information construction work in tertiary class-A public hospitals as well as the current problems and countermeasures in hospital information construction work to provide strategic support for the next high-quality development of medical informatization.

## Methods

### General Information

The study was conducted among the members of the Chen Xiaoping Academician Workstation of Hepatobiliary and Pancreatic Surgery. In China, there is a hospital rating system to evaluate the public hospital by 3 levels and 6 classes. Medical services and management, medical quality and safety, and medical technology level and efficiency are the 3 main assessment targets [20]. Tertiary class-A is the highest level of the comprehensive hospitals according to the “3 levels and 6 classes” assessment rules, meaning that it should have a high level of hospital information construction. Hence, this study included all tertiary class-A public hospitals among all members of the academician workstation. The questionnaires used in study were distributed by email in April 2020 and were collected in July 2020. A total of 24 questionnaires were distributed and 23 were collected. Among the 23 tertiary class-A public hospitals who provided feedback with the questionnaires, 3 were from East China, 5 were from Midland China, 3 were from South China, 6 were from West China, and 6 were from North China. The respondent at each hospital was the department director, vice dean, or dean.

### Method of Investigation

This study was conducted using a self-designed questionnaire. The questionnaire was referred to in a previous study [21] and discussed, revised, and finalized by study groups to make it more suitable for this study. The questionnaire included questions around 4 latitudes: configuration of hospital information department, configuration of hospital information system, configuration and practical application of hospital internet service, and the satisfaction of hospital information construction. Furthermore, interviews were also conducted with the respondents in this study for requirements analysis.

### Ethics Approval

This study was approved by the ethics committee of Tongji Hospital (TJ-IRB20220103). There were no active participants in the study data, and the ethics committee waived the requirement for informed consent. All methods in this study were carried out in accordance with relevant guidelines and regulations.

### Statistical Analysis

A database for the information obtained from the collected questionnaires was constructed using EpiData software (version 3.1; EpiData Association), and the data from each questionnaire were recorded twice by 2 staff members working together to ensure that the data were recorded without any typing errors. Data were presented by frequencies and percentages, and analyses were performed using SPSS Statistics software (version 21; IBM Corp).

## Results

Table 1 shows the configuration of the information departments in the hospitals included in this study. The results indicated that the 23 hospitals had established independent information departments with special information directors for management, of which 85% (n=19) were directly under the responsibility of vice presidents and above, and a few (n=4, 17%) were partly managed by other departments (eg, equipment department and medical department). The direct management of the information department by senior hospital leaders (vice presidents and above) can give hospitals more power and space for the development of information systems, suggesting that China’s tertiary class-A public hospitals give more importance to the construction of hospital information technology. Additionally, 21 (91%) of the 23 hospitals had hired professional IT workers, accounting for more than 90% of the study sample. However, only 16 (70%) hospitals employed their owner IT staff to routinely maintain and operate information technology projects. In general, in China’s effort to facilitate information construction in tertiary class-A public hospitals, information technology talent was still limited.

In recent years, the policies related to medical informatization have clarified the evaluation goals of EMR grading and management [22] and emphasized that the construction of hospital information with EMR should strengthen the standardization and standardized management of clinical data. As can be seen in Table 2, first, the EMR system, as the core of the rating, should be constructed, with all hospitals being equipped with an EMR system. Second, the hospital information management system (HIS), picture archiving and communication system (PACS), and laboratory information system (LIS) each had a coverage rate of more than 90%. Third, the construction of radiography information system (RIS) was slightly weaker, with only 20 (87%) out of the 23 hospitals being equipped with this system. Overall, the construction of hospital information systems in the tertiary class-A public hospitals was good, as there were 17 hospitals equipped all systems, but the remaining 6 hospitals, which are located in second- and third-tier cities, lacked 1 or 2 of the information systems listed in Table 2. Therefore, the construction of hospital information system still needs to be further improved.

Table 3 shows the list of the configuration and application of hospital internet services. From the table, we can see that in terms of hospital internet service configuration, web-based appointment registration and EMR services were the 2 most widely configured, with both exceeding 95%, followed by external promotion and introduction (n=19, 83%), telework and portal application (n=18, 78%), web-based medical consultation (n=16, 70%), and business-to-business transaction (n=14, 61%). Web-based health assessment services were at a low level, covering only about 52% (n=12). In all, 9 hospitals, which are all located in provincial capitals, had equipped all the web-based applications listed in Table 3. For the internet service applications, this study investigated the number of web-based registrations, and 26% (n=6), 26% (n=6), and 30% (n=7) of the registrations were in the range of 5% to 20%, 20% to 50%, and more than 50%, respectively, and only 17% (n=4) of the hospitals had fewer than 5% of web-based registrations. We screened the location distribution of the different levels of web-based registrations: the 4 hospitals that were below 5% are in second- and third-tier cities, and the 7 hospitals that were above 50% are all located in first-tier cities and provincial capitals. Overall, the application of web-based registration services was fair, and in the context of the outbreak of COVID-19, web-based registrations and other services will have a greater demand for development.

Table 4 shows the results of the satisfaction of hospital medical information construction. In all, 83% (19/23) of medical workers believed that their hospital focused on the development of hospital information, but only 61% (14/23) were satisfied with the development of hospital information. Overall, the medical workers were less satisfied with the hospital IT staff, with the satisfaction rate being 43% (10/23). Moreover, 65% (15/23) of the medical workers believed that their hospitals had abundant medical information, but the utilization rate of this medical information was very low.

**Table 1:** Configuration of hospital information department.

Item			Hospital (N=23), n (%)
Has your hospital set up a specialized information department? (yes)			23 (100)
Has your hospital set up a special information director? (yes)			23 (100)
**Who is the special information director?**			
	Vice presidents and above	19 (85)	
	Other	4 (17)	
Does your hospital have IT workers? (yes)			21 (91)
**How is the daily maintenance of the hospital information project completed?**			
	Outsourcing	7 (30)	
	Hospital IT staff	16 (70)	

**Table 2:** Configuration of hospital information system.

Item	Hospital (N=23), n (%)
HIS^a^	22 (96)
LIS^b^	21 (91)
EMR^c^	23 (100)
PACS^d^	22 (96)
RIS^e^	20 (87)

^a^HIS: hospital information management system.

^b^LIS: laboratory information system.

^c^EMR: electronic medical record.

^d^PACS: picture archiving and communication system.

^e^RIS: radiography information system.

**Table 3:** Configuration and practical application of hospital internet service.

Item		Hospital (N=23), n (%)
**Internet service**		
	External promotion and introduction	19 (83)
	Web-based medical consultation	16 (70)
	Web-based hospital appointment register	22 (96)
	Web-based health assessment	12 (52)
	EMR^a^ services	22 (96)
	B2B^b^ exchange	14 (61)
	Telework and portal application	18 (78)
**Proportion of** **hospital appointment register**		
	Below 5%	4 (17)
	5% to 20%	6 (26)
	20% to 50%	6 (26)
	50% above	7 (30)

^a^EMR: electronic medical record.

^b^B2B: business to business.

**Table 4:** Satisfaction of hospital medical information construction.

Item		Value, n (%)
**Do you think medical informatization construction is valued in your hospital? (n=23)**		
	None	0 (0)
	Relatively valued	1 (4)
	Neutral	3 (13)
	Valued	11 (48)
	Very valued	8 (35)
**Are you satisfied with your hospital’s medical informatization? (n=23)**		
	Very unsatisfied	0 (0)
	Unsatisfied	2 (9)
	Neutral	7 (30)
	Satisfied	9 (39)
	Very satisfied	5 (22)
**Does your hospital IT staff satisfy the demands? (n=21)**		
	Very unsatisfied	0 (0)
	Unsatisfied	1 (4)
	Neutral	10 (44)
	Satisfied	8 (35)
	Very satisfied	2 (9)
**Do you think the utilization rate of abundant medical information in your hospital is very low? (n=23)**		
	Very low	2 (9)
	Relatively low	4 (17)
	Neutral	9 (39)
	Relatively high	5 (22)
	Very high	3 (13)

## Discussion

### Principal Findings

Driven by the national policies, information construction has become one of the priorities of hospitals’ daily work. In our research, all hospitals had set up independent information department, which were almost all led by a senior leader of hospitals. However, only 70% hospitals in our study had built an IT team to construct and maintain the information projects at the hospital. Others had outsourced to other companies.

All hospitals were equipped with an EMR system in our study. This result is not surprising, since EMR is a very important evaluation index in China’s hospital ranking system. Besides, the utility rates of HIS and PACS were also very high (over 95%). The utility rates of LIS and RIS were a little lower than abovementioned systems, but they all exceeded 90%. Overall, the infrastructural construction of hospital information was relatively good.

In the aspect of hospital internet service, web-based appointment registration and EMR service were the most widely used. Other internet services such as external promotion and introduction, telework and portal application, web-based medical consultation, and business-to-business transaction were gradually developed and applied. In addition, we also found that hospitals in provincial capitals had a variety of internet services with higher utility rates.

Notwithstanding, the infrastructural construction of hospital information was satisfactory. However, the hospitals’ staff were not satisfied with the data utility. Half of them in our study thought that the huge scientific and economic value hidden in the massive data stored in hospital information systems had barely been mined.

### Implications

In China, the distribution and allocation of medical resources are incredibly unbalanced: almost 80% of medical resources and patients are concentrated in large hospitals and 20% are in primary care institutions [23]. There is a similar trend in the construction of hospital information. Developed regions, first-tier cities, and top-tier hospitals are sitting on high-quality medical resources and have an early start and large investment in information technology construction, with enough funds and human resources [24]. This is a common problem in transitional countries. For over 20 years, the failures of medical informatization in transitional countries were mainly due to the infrastructural gaps [25]. According to the investigation results of the present study, the overall information system of large hospitals was relatively complete, and the hospital information systems, such as HIS, LIS, EMR, PACS, and RIS, were also well equipped. Based on the interviews, the next steps of information construction in large hospitals are in relation to the following aspects: higher-rated EMR system, widely interconnected information platform, more convenient and intelligent internet services, translating big medical data into valuable applications with cutting-edge technologies, and so on. In contrast, primary care institutions have limited resources in this area, and their level of investment and construction of information technology development is far lower than that of large hospitals. Noticeably, the gap between top-tier hospitals and primary care institutions will hinder the data interaction and limit the insights and extensive application of big medical data.

Medical information interconnection is a process of fusing multisource data into big data, which have great scientific and commercial value. However, the results of the present study showed that over 65% of the medical workers believed that the abundant medical data in their system was not utilized well. At present, most hospital information system still stay at computerizing the paperwork and data integration. In our interviews, most of the IT staff in the information department focused on the maintenance of the information system. The optimization of workflow is most focused on the quality control of EMR. Data mining is predominantly reserved for administrative management. However, with technology changing especially rapidly, the high application values of big data are being more and more realized. Medical scientists have foreseen the potential value of big medical data stored in hospital information systems [26]. Unfortunately, current hospital information systems cannot support this use for scientific research effectively, and medical scientists are always absent from hospital information construction. This is a common problem in the construction of medical informatization, which will seriously restrict further development of medical informatization [27].

In fact, the construction of medical information technology involves a wide range of aspects. The talent involved in the process of information technology construction needs to have a hospital-related medical background, be familiar with the hospital workflow, and have a theoretical knowledge on information technology [28]. Having this type of complex talent and clinical worker that coparticipate in the hospital information technology construction will certainly be able to broaden the insights into the information contained behind the massive data deposited in the hospital information system. This will further improve the use of medical data and build a synergistic system of “information plus” and clinical care, personnel training, scientific research, and the transformation of experimental findings, so that the hospital information construction meets the actual needs of clinical research. Moreover, this will meet the information data support for administrative decision-making and empower the improvement of medical service quality [29]. Several research had revealed the high willingness of medical students to receive medical informatics training in their study period (90% for American, 70% for German, 83% for South Korean, and 78% for British students) [30-32]. Therefore, we should actively train a group of composite talents with both medical and information backgrounds and encourage clinical workers to actively participate in the hospital information construction work through various forms, such as title evaluation, achievement recognition, and performance rewards.

The other very prominent problem in overall hospital information construction is the lack of Uniform Data Standards. There is an international series standard, which has been in development since the 1980s—Health Level 7 (HL7) Fast Healthcare Interoperability Resources (FHIR) standard—that was released in 2019 [33]. The HL7 FHIR standard includes a unified description and understanding of different data to facilitate rapid exchange and understanding of data from different sources and in different system. It can be said that HL7 FHIR is a facilitator of information exchange between different systems. However, FHIR is not widely used in China at present. Only a few hospitals as a pilot hospital have built relevant systems and platforms by FHIR [34]. In the early stage of information technology construction, almost no top-level planning and overall coordination were observed, and different manufacturers in the development and production of information technology systems used different standards in China. At present, most information platforms have been built according to the needs of each medical institution. The conceptual system and data type of medical institutions are different, and the same data may be recorded by multiple heterogeneous systems at the same time, which often leads to conflict and redundancy in the process of information interaction. The system data model used in medical units is not open, so data exchange and sharing cannot be realized, which affects the extraction and use of data and challenges the information exchange between systems in the medical field. The poor compatibility between the different information technology systems caused many difficulties in the completion of information interoperability with other hospitals or institutions.

The specialist medical information concept is being born at the right moment. In the process of specialist medical information construction, another level of data standard inconsistency is present, that is, the data recording standards are not uniform among the different medical institutions. Although the EMR can now enter part of the objective data in tabular form, most of the information related to disease development and treatment is still recorded using large texts, such as first course, daily note, surgical records, and so on, in the EMR. In retrospective studies, adequate human resources are required to extract valid data from medical records, and missing information in the extracted data is inevitable. In multicenter retrospective studies, different centers may have missing data due to different medical record writing habits. Moreover, the missing data will cause a lot of problems and greatly reduce the validity of the research results. The construction of information technology can save the process of manual extraction of information, that is, the poststructuring of textual information through natural language processing technology [35], but the lack of information cannot be compensated if the doctor does not write the information into the EMR. Thus, in the process of building medical information technology, according to the type of standard for data collection, what information should be recorded in the EMR? This is the most important point of the problem that must be solved, and these standards have not been established yet.

At present, the EMR is mostly a general EMR that was upgraded from the perspective of hospital-wide application. In recent years, the concept of specialized EMR has gradually emerged [36,37]. The specialized EMR is an integrated system of medicine, management, research, and education based on the general EMR and combined with specialized diagnosis and treatment process, so as to better serve the specialized diagnosis and treatment behavior and improve patient satisfaction. For the construction of specialized EMR, it is necessary to establish a standard data set that combines the characteristics of specialized medical treatment, and under the guidance of this standard data set, the general EMR can be renovated and upgraded in a targeted manner. The construction of the specialized medical data standards is not only for creating specialized EMR services but also for laying a solid data foundation for future data mining and specialized information application. With the rapid development and common application of big data mining technology in recent years, the application of medical big data, such as clinical auxiliary decision-making, clinical research and development, and medical intelligence, has already had a more mature technical environment, and what is lacking now is high-quality medical big data. Therefore, in the process of hospital information construction, the problems related to the construction of specialized medical data standards need to be addressed.

### Limitations

A limitation in our study is the small sample size, which may lead to being underpowered for inferential analyses. However, our findings indeed exposed existing problems in medical information construction. The interviews with the responsible persons involved in our study also provided valuable insight to improve the existing hospital medical information systems. Accordingly, we believe our findings are objective, and it calls for further study in larger samples.

### Conclusions

Hospital medical information construction has always been a challenging issue in transitional countries. In China, although the infrastructural information construction of medical of tertiary class-A public hospitals has been completed, various problems are still present, hindering the development of hospital medical information construction. The resolution to improve the overall level of hospital information construction in transitional countries still requires long-term endeavors. Support for the construction of information technology in primary care institutions should be increased to balance the level of development of information construction in medical institutions at all levels; the training of complex talent with both IT technology and medical backgrounds should be emphasized; and specialized disease information standards should be developed to lay a solid data foundation for data use and improve the use of medical big data.

## Abbreviations

EMR: electronic medical record
FHIR: Fast Healthcare Interoperability Resources
HIS: hospital information management system
HL7: Health Level 7
LIS: laboratory information system
PACS: picture archiving and communication system
RIS: radiography information system

## Data Availability

The data sets generated and analyzed during the current study are not publicly available due the need of the other hospitals that contributed to this study to carry out other research but are available from the corresponding author on reasonable request.
